# Cancer and Noncancer Mortality Among American Seafood Workers

**DOI:** 10.2188/jea.JE20100147

**Published:** 2011-05-05

**Authors:** Eric S. Johnson, Mohammed F Faramawi, Macodu Sall, Kyung-Mee Choi

**Affiliations:** 1 Professor and Chairman, Epidemiology Department, School of Public Health, University of North Texas Health Science Center, Fort Worth, TX; 2 Assistant Professor, Epidemiology Department, School of Public Health, University of North Texas Health Science Center, Fort Worth, TX; 3 School of Public Health, Tulane University, New Orleans, LA; 4 Assistant Professor, Environmental & Occupational Health Sciences Department, School of Public Health, University of North Texas Health Science Center, Fort Worth, TX

**Keywords:** mortality, occupation, neoplasms

## Abstract

**Background:**

Few studies have investigated mortality in seafood workers worldwide, and no such study has been conducted in the United States. The objective of this study was to investigate mortality in American seafood workers.

**Methods:**

The study population was derived from 4 states and consisted of 4116 subjects who worked mainly in seafood processing plants. They were followed up from 1966 to 2003. Standardized mortality ratios (SMRs) and proportional mortality ratios (PMRs) were estimated, using the US general population for comparison.

**Results:**

About 45% of the cohort was born after 1949. A total of 788 deaths were recorded; 53% of the decedents were female, and 88% were white. The SMRs for stomach cancer and disorders of the thyroid gland in the cohort as a whole were 2.1 (95% confidence interval [CI], 1.1–3.8) and 6.1 (95% CI 1.3–18.0), respectively. The SMRs for breast cancer, and occlusion/stenosis of the pre-cerebral/cerebral arteries in the cohort as a whole were 0.5 (95% CI, 0.3–0.9) and 0.5 (95% CI, 0.2–0.8), respectively. The SMR for ischemic heart disease in white females was 0.8 (95% CI, 0.6–0.9).

**Conclusions:**

This cohort had excess deaths from stomach cancer and disorders of the thyroid gland, and deficit of deaths from breast cancer, stroke and ischemic heart disease. The significance of these findings is unknown, especially as less than 20% of the cohort were deceased. Nevertheless, the cohort is unique and important, and further follow-up may shed more light on mortality patterns in this occupational group.

## INTRODUCTION

The seafood industry has experienced tremendous growth in recent years.^1^ The Food and Agriculture Organization (FAO) estimates that the number of people working in the fishing industry, aquaculture, and related activities worldwide has doubled to 28.5 million since 1970.^1^ The industry is well known to be one of the most hazardous, with high rates of fatal accidents.^1^ However, few studies have reported on cause-specific mortality in this occupational group.^2^^–^^4^ To our knowledge, no data have been published on cause-specific mortality in American seafood workers. Therefore, we investigated mortality in a cohort of seafood workers in the United States.

## METHODS

The study population (*n* = 4116) was defined as all people who worked in seafood occupations anytime between 1966 and 1990 and were members of the United Food and Commercial Workers (UFCW) International Pension Fund. It included employees of 7 seafood companies in the northern United States who worked on fishing vessels or in wholesale processing plants or warehouses where various fresh or frozen seafood (fish, shrimp, crab, lobster, clam, etc.) were cut, cleaned, scaled, gutted, filleted, packed in containers and crates, or stored. Processing activities also include battering, breading, and frying of various types of fish and other seafood, as well as production of shrimp paste. The cohort also included subjects who worked in seafood retail stores and a seafood restaurant. Study subjects were followed up for mortality from 1 January 1966 to 31 December 2003, during which 788 deaths occurred in the cohort. The cause of death was coded according to the Ninth Revision of the International Classification of Diseases (ICD-9).

Methods of follow-up included use of national databases such as the National Death Index (NDI) which records all deaths in the country, Social Security Administration, state Departments of Vital Records, state Departments of Motor Vehicles, personal contact by telephone and mail, and internet tracing methods. Pension Benefit Information Inc, a private company, was also used to identify deceased persons. This company matches subjects against US death records for all years from the 1800s to the present, using information received from various government databases such as the Social Security Administration, Health Care Financing Administration, and state Departments of Vital Records, as well as the Civil Service Commission, Railroad Retirement Board, and the Department of Defense. Because we carried out a comprehensive search for death records (it has been shown for example that NDI will identify greater than 98% to 99% of deaths if matching information is good), subjects for whom no death records could be identified at the end of follow-up were assumed to be alive at the end of study.

Information on date of birth was missing for 39 seafood workers (0.9%) whose vital status was also unknown. Rather than excluding such persons from the analysis, their date of birth was imputed, based on the median year of birth recorded for workers with known dates of birth who joined the union the same year. Thus, if a member with an unknown date of birth joined the union in 1975, she was assigned as her year of birth the median year of birth for persons with known birth dates who joined the union in that particular year. This crude measure was deemed to be associated with minimal bias, since the effect on total person-years would be negligible and non-significant.

Statistical analyses involved estimation of standardized mortality ratios (SMRs), stratified by age, calendar time, sex, race and processing plant, using the US general population as the comparison group. Analysis was conducted using the OCMAP Plus software package from the University of Pittsburgh, USA. US mortality rates were purchased from the University of Pittsburgh. Information on race was available only for decedents with a known cause of death. Therefore, to perform the SMR analyses, race was artificially assigned at random to each individual without a known cause of death, based on the racial distribution of decedents with known race. In a similar study of UFCW workers in the meat industry, the racial distribution of deceased subjects was found to be no different from that of a sample of current workers representing more than half the union membership.^5^ The cohort was stratified by plant and then further stratified into 4 subgroups by race and sex (black males, black females, white males, white females), after which each of these groups was stratified according to age (5-year intervals) and calendar year at entry into the cohort (5-year intervals). Person-years were accumulated from the date the company joined the union for those subjects who were already employed in the company by that date. For those who started working after their company had joined the union, accumulation of their person-years commenced on the date of union membership, which was virtually the same as their date of hire, since membership in the union was compulsory from the first day of employment. Person-years were enumerated up to the date of death or date of termination of the study on 31 December 2003, whichever was earlier. Expected deaths were derived by multiplying the person-years in each cell by the corresponding sex-, race-, calendar year-, and age-specific mortality rate for the US general population. Observed and expected deaths for each cell were summed over all ages and calendar years, and over all strata, and the SMR was estimated as the total observed number of deaths divided by the total expected. The 95% confidence intervals for the SMRs were calculated according to a simple exact method that links the Poisson and chi-square distributions.^6^ A similar method of stratification was used to estimate the PMR, except that for each cell in the study population, the corresponding proportion of all deaths due to a given cause in the US population was multiplied by the total number of deaths in that cell to obtain the expected number of deaths. The ratio of observed deaths in the cell to expected deaths is the PMR. Observed and expected deaths for each cell were summed over all ages and calendar years, and over all strata, and the PMR was estimated as the total observed number of deaths divided by the total number of expected deaths.

The protocol for this study was approved by the University of North Texas Health Science Center Institutional Review Board.

## RESULTS

Table 1 shows the location of the seafood companies, their activities, and the number of workers. Most subjects worked in seafood processing plants. Table 2 shows the year-of-birth distribution of the cohort; about half the cohort members were born in 1950 or later. A total of 788 deaths were recorded for the 4116 seafood workers; 88% of the decedents were white and 53% female. The average number of person-years contributed by each subject was 25.2 (Table 3). Table 4 shows the main results regarding causes of death for which more than 1 death occurred in the seafood cohort, and for which a statistically significant SMR was observed in any race/sex subgroup or in the entire seafood cohort. All-cause mortality was lower than that of the general population in all race/sex subgroups except white males. The SMR for all malignant neoplasms was not significantly elevated in the cohort as a whole, or in any race/sex subgroup, although it was significantly lower in nonwhites as a group. Regarding specific cancers, the overall risk of death from stomach cancer was elevated in the cohort, but not significantly so (SMR, 2.0). Elevated SMRs for stomach cancer were recorded for all race/sex subgroups except nonwhite men, and the SMR of 2.8 for stomach cancer in women was statistically significant. A significant deficit in deaths from breast cancer was observed in women. For non-cancer deaths, mortality from disorders of the thyroid gland was significantly increased in the cohort and was mainly confined to whites. The SMRs for ischemic heart disease and occlusion/stenosis of the pre-cerebral and cerebral arteries were also significantly lower among female seafood workers compared with the US general population. The risk of death from transport accidents was not increased, and there were no deaths from water transport accidents (not shown). As is evident in Table 4, the PMR and SMR results were almost identical.

**Table 1.** Company location, year unionized, description of plants activities and number of workers

Company	Location of company	Year unionized	Description of activities	No. of subjects
1	Gloucester, MA	1973	Workers in fishing vessels, wholesale processing plants, and retail fish stores	1765

2	Gloucester, MA	1973, 1973 1986, 1986	Workers in 4 processing plants belonging to the same company that processed frozen fish and other frozen seafood products	875

3	Gloucester, MA	1973	Workers in a processing plant and warehouse that handled only fish. Activities include cutting, scaling, gutting, and packing	162

4	Chicago, IL	1966	Workers in 2 retail stores, plus a processing plant and warehouse where workers cut, clean, and pack fish such as swordfish, sea bass, marlin, scrod, snapper, whitefish, and other seafood, and make products such as lobster and shrimp bisque	153

5	Chicago, IL	1966	Workers in a processing plant and warehouse that handled fish, clams, crabs, lobsters and shrimps	150

6	Miami, FL	1977	Workers in a processing plant that handled shrimp and shrimp-based products ranging from shrimp paste, to frozen breaded and fried shrimps.	230

7	Cleveland, OH	1969	Workers in a processing plant and warehouse that handled all types of seafood, from shrimp, lobster, clam, crab, whiting, cod, haddock, catfish, and halibut; activities include battering and breading fish, and packaging into containers	781

	Total			4116

**Table 2.** Year-of-birth distribution of seafood workers

Interval	No. of subjects	Percentage	Cumulative percentage
1870–1900	3	0.1	0.1
1900–1910	76	1.9	2.0
1910–1920	343	8.3	10.3
1920–1930	494	12.0	22.3
1930–1940	574	14.0	36.3
1940–1950	765	18.6	54.8
1950–1960	1399	34.0	88.8
After 1960	462	11.2	100.0
Total	4116	100.0	

**Table 3.** Number of subjects, person-years at risk, and number of deaths among seafood workers, by race and sex

Race	Males			Females			All		
		
	No.	Person-years	Deaths	No.	Person-years	Deaths	No.	Person-years	Deaths
White	1619	40 016.1	329	2050	52 491.3	363	3669	92 507.4	692
Non-white	236	5976.2	44	211	5363.0	52	447	11 339.1	96
Total	1855	45 992.3	373	2261	57 854.3	415	4116	103 846.5	788

The racial distributions of no. of subjects and person-years were imputed from those of deceased subjects with known race.

**Table 4.** Cause-specific mortality in seafood workers (1966–2003): standardized mortality ratios and proportional mortality ratios

Cause of death	Seafood workers								

	Non-white males	White males	All males	Non-white females	White females	All females	All non- whites	All whites	All groups
								
	Obs SMR 95% CI PMR 95% CI [*n* = 236]	Obs SMR 95% CI PMR 95% CI [*n* = 1619]	Obs SMR 95% CI PMR 95% CI [*n* = 1855]	Obs SMR 95% CI PMR 95% CI [*n* = 211]	Obs SMR 95% CI PMR 95% CI [*n* = 2050]	Obs SMR 95% CI PMR 95% CI [*n* = 2261]	Obs SMR 95% CI PMR 95% CI [*n* = 447]	Obs SMR 95% CI PMR 95% CI [*n* = 3669]	Obs SMR 95% CI PMR 95% CI [*n* = 4116]
All malignant neoplasms	7 0.5 (0.2–1.1) 0.7 (0.4–1.3)	65 0.9 (0.7–1.2) 0.8 (0.7–1.0)	72 0.8 (0.7–1.1) 0.8 (0.7–1.0)*	11 0.7 (0.3–1.2) 0.8 (0.5–1.4)	123 1.0 (0.8–1.2) 1.1 (1.0–1.3)	134 1.0 (0.8–1.1) 1.1 (0.9–1.3)	18 0.6 (0.4–1.0)* 0.8 (0.5–1.2)	188 1.0 (0.8–1.1) 1.0 (0.9–1.1)	206 0.9 (0.8–1.0) 1.0 (0.9–1.1)

Malignant neoplasm of the stomach	0 — 0 —	3 1.5 (0.3–4.3) 1.4 (0.4–4.2)	3 1.1 (0.2–3.3) 1.1 (0.4–3.5)	2 3.6 (0.4–13.1) 4.3 (1.2–15.1)*	6 2.6 (1.0–5.7) 3.1 (1.4–6.5)*	8 2.8 (1.2–5.5)* 3.3 (1.7–6.3)*	2 1.7 (0.2–6.2) 2.1 (0.6–8.2)	9 2.1 (1.0–4.0) 2.2 (1.1–4.1)*	11 2.0 (1.0–3.6) 2.2 (1.2–3.8)*

Malignant neoplasm of the breast	0 — 0 —	0 — 0 —	0 — 0 —	0 — 0 —	14 0.6 (0.3–1.0) 0.7 (0.4–1.1)	14 0.5 (0.30–0.9)* 0.6 (0.4–1.0)	0 — 0 —	14 0.6 (0.3–1.0) 0.7 (0.4–1.1)	14 0.5 (0.3–0.9)* 0.6 (0.4–1.0)

Disorders of the thyroid gland	0 — —	2 35.0 (4.2–126.6) 32.7 (13.7–78.2)*	2 27.6 (3.3–100.0) 27.7 (11.2–68.7)*	0 — —	1 2.7 (0.1–15.2) 3.4 (0.5–21.6)	1 2.4 (0.1–13.4) 3.0 (0.5–19.3)	0 — —	3 7.1 (1.5–20.7)* 8.5 (3.3–21.8)*	3 6.1 (1.3–18.0)* 7.4 (2.8–19.3)*

Ischemic heart disease	10 1.0 (0.5–1.9) 1.4 (0.8–2.4)	81 1.1 (0.9–1.3) 1.1 (0.9–1.3)	91 1.1 (0.9–1.4) 1.1 (0.9–1.3)	10 0.9 (0.4–1.7) 1.0 (0.6–1.7)	67 0.8 (0.6–1.0)* 0.9 (0.8–1.2)	77 0.8 (0.6–1.0)* 0.9 (0.8–1.2)	20 1.0 (0.6–1.5) 1.2 (0.8–1.7)	148 0.9 (0.8–1.1) 1.0 (0.9–1.2)	168 0.9 (0.8–1.1) 1.0 (0.9–1.2)

Occlusion/stenosis of pre-cerebral & cerebral arteries	2 1.7 (0.2–6.2) 2.1 (0.6–7.7)	4 0.8 (0.2–2.2) 0.8 (0.3–2.1)	3 1.0 (0.4–2.2) 1.0 (0.5–2.2)	1 0.5 (0.0–2.9) 0.4 (0.1–2.5)	4 0.3 (0.1–0.7)* 0.3 (0.1–0.8)*	5 0.3 (0.1–0.7)* 0.3 (0.2–0.8)*	3 1.0 (0.2–2.8) 0.9 (0.3–2.6)	8 0.4 (0.2–0.8)* 0.5 (0.2–0.9)*	11 0.5 (0.2–0.9)* 0.5 (0.3–0.9)*

Transport accidents	0 — 0 —	9 0.7 (0.3–1.4) 0.6 (0.3–1.0)	9 0.6 (0.3–1.2) 0.5 (0.3–1.0)*	1 1.7 (0.0–9.6) 2.6 (0.4–17.1)	1 0.2 (0.0–0.9) 0.2 (0.0–1.3)	2 0.3 (0.0–1.1) 0.4 (0.1–1.5)	1 0.4 (0.0–2.1) 0.7 (0.1–4.4)	10 0.5 (0.3–.1.0) 0.5 (0.3–0.9)	11 0.5 (0.3–0.9) 0.5 (0.3–0.9)

All-cause mortality	44 0.7 (0.5–1.0)*	329 1.1 (1.0–1.2)	373 1.0 (0.9–1.2)	52 0.9 (0.6–1.1)	363 0.8 (0.8–0.9)*	415 0.8 (0.8–0.9)*	96 0.8 (0.6–1.0)*	692 0.9 (0.9–1.0)	788 0.9 (0.9–1.0)*

Abbreviations: Obs, observed; SMR, standardized mortality ratio; PMR, proportional mortality ratio.

There are no PMR estimates for all-cause mortality.

*Statistically significant at the 95% level of confidence.

— Could not be calculated.

## DISCUSSION

The absence of major differences between the SMR results in which race was imputed for nondeceased subjects, and the PMR results which were based on complete information on race, is reassuring and indicates that no serious bias resulted from imputing race in the SMR analyses. With the exception of white males, seafood workers in the current study tend to exhibit the healthy worker effect, ie, lower overall mortality than that of the general population. It is not clear why decreased mortality was not observed in white men.

An excess of stomach cancer was recorded in all race/sex subgroups except nonwhite males, although the small sample size of this subgroup may be partly responsible for the apparent absence of risk. An excess occurrence of stomach cancer was previously reported in Chinese fishermen in Singapore, and all cases were divers.^7^ A study of Swedish fishermen reported a significant excess incidence of stomach cancer in fishermen on the east coast but not on the west coast.^4^ Thus our findings for stomach cancer are consistent with the results of those 2 studies. In addition, in the Swedish study, the wives of east coast fishermen had a significantly increased risk of stomach cancer, while the wives of west coast fishermen had an elevated risk that was not statistically significant.^4^ East coast fishermen and their wives had a higher intake of fatty fish than those on the west coast. They also had increased concentrations of persistent organochlorine pollutants in their blood, while west coast fishermen and their wives did not.^7^^,^^8^ Other reports have noted significantly higher fish consumption among seafood workers as compared with the general population, based on information from dietary interviews.^4^^,^^9^ These reports raise the possibility that the excess of stomach cancer reported in seafood workers could be partly explained by dietary factors rather than, or in addition to, occupational factors. Unfortunately, we have no evidence that seafood workers in the United States consume more seafood than the US general population. A possible explanation for the excess is that seafood workers generally have unhealthy lifestyles.^9^ Previous studies have reported high levels of smoking and alcohol intake in the fisheries sector.^9^^,^^10^ Smoking and alcohol intake are known risk factors for many cancers, including stomach cancer, although the association with alcohol is inconsistent.^11^ Another possibility is that seafood workers may have higher dietary intakes of salted fish, “rotted” seafood, or both. Seafood salting increases the content of nitrates in the diet.^7^ Furthermore, rotted seafood could lead to bacterial contamination of the stomach. Nitrates could be subsequently reduced to nitrites in the gastrointestinal tract, and the bacterial flora or amines present in food could lead to conversion of nitrites to carcinogenic N-nitroso compounds.^7^ It is also possible that seafood workers in trawlers and fishing boats are exposed to carcinogenic diesel fumes or asbestos, although these exposures are not usually associated with stomach cancer.^12^^–^^17^

In the present study, the lower occurrence of breast cancer among female seafood workers could be partly attributed to their high fish intake, as mentioned above. The omega-3 fatty acids in fatty fish, have consistently been shown to retard the growth of breast cancer cells *in vitro* and in animal experiments.^18^^–^^20^ However, the reported association between fish consumption and breast cancer in women has not been consistent, as some epidemiologic studies have found a significant inverse association, while others have not.^21^^–^^25^

It is unclear why an excess of disorders of the thyroid gland was observed in this cohort. Thyroid disorders can be caused by excess iodine intake, which can result from high consumption of seafood rich in iodine.^26^^,^^27^ Thus the excess occurrence of thyroid diseases in workers could be related to nonoccupational rather than occupational factors. The reason why the excess of thyroid diseases was restricted to whites is also not known, but the small sample size of nonwhites is a possible explanation.

Our findings indicate that ischemic heart disease and stenosis of the pre-cerebral and cerebral vessels were less frequent among female sea-food workers in this cohort. In the Swedish study described above,^4^ the SMR for ischemic heart disease as in our cohort, was significantly depressed in west coast fishermen and their wives, but not in those on the east coast. Similarly, the SMR for cerebrovascular accidents was depressed in west coast fishermen, but not in east coast fishermen, although the reduction was not significant.^4^ Frequent consumption of seafood, particularly fish, has been associated with reduced stroke and coronary heart disease mortality in several reports.^28^^–^^33^ Thus, the present observations although somewhat inconsistent, suggest that dietary factors such as increased seafood consumption play a role in the occurrence of cardiovascular and cerebrovascular diseases in seafood workers.

Fishermen in fishing boats are known to be at high risk of water transportation accidents.^1^ No deaths from water transportation accidents were recorded in this cohort, with 0.5 expected. The low statistical power of the study may be partly responsible for this finding. In addition, most seafood workers in this cohort were employed in processing plants, and very few were likely to have worked in fishing vessels as fishermen.

This study should be interpreted in the context of its limitations. Firstly, as is the case in many retrospective cohort mortality studies, information was missing on specific occupational exposures and nonoccupational factors such as smoking and seafood consumption. Thus the specific cause(s) responsible for the excesses and deficits cannot be identified in this type of study. Secondly, the statistical power was low, and important associations could have been missed. Thirdly, multiple comparisons were made, as we examined in total 185 separate causes of death^34^ in each of several race/sex subgroups; thus, some of the observed associations could have been due to chance, especially as only a small number of deaths were involved in some instances. Finally, it was not possible to identify and analyze in detail the data separately for fishermen who worked in fishing vessels, workers in retail stores and workers in processing plants, because company records did not precisely distinguish these groups. A limited examination however, indicated that the excess of stomach cancer appeared to occur in all these groups.

There was no excess of the cancers known to be associated with the specific carcinogenic exposures that may have occurred in this occupational group. These exposures include oncogenic viruses that cause tumors in fish,^35^ and polycyclic aromatic hydrocarbons and heterocyclic amines that are formed during cooking or smoking of seafood.^36^^,^^37^

Interestingly, no association between these occupational exposures and stomach cancer has been previously observed. Indeed, we observed no excess stomach cancer in our concurrent investigation of 3 poultry cohorts from the same Pension Fund,^38^^–^^40^ and none of the cancers that were in excess in the poultry cohorts were in excess in the seafood cohort. Similarly, the findings for breast cancer and stroke in this seafood cohort were not observed in the poultry cohorts. Thus the findings in this cohort of seafood workers appear unique.

The study has some advantages. Firstly, to our knowledge, this is one of the largest studies of seafood workers to date, particularly of workers in processing plants, and the only study of this occupational group in the United States. Secondly, the definition of the cohort was uniquely comprehensive. Because of the exceptional union recordkeeping system, all employees (even those who worked for a brief period, such as a few days) had a personal record, making selection bias unlikely. Thirdly, because the present investigation was a cohort study, a temporal relationship between the exposure surrogate (affiliation with the cohort) and the outcome (disease-specific mortality) could be established.

In summary, the findings of this study are preliminary and hypothesis-generating and draw attention to seafood workers in the United States. Further follow-up and case-control studies nested within this cohort are encouraged. Nested case-control studies will allow for more comprehensive assessment of exposures and better control of occupational and nonoccupational confounding factors.
